# Possible Role of the Glycogen Synthase Kinase-3 Signaling Pathway in Trimethyltin-Induced Hippocampal Neurodegeneration in Mice

**DOI:** 10.1371/journal.pone.0070356

**Published:** 2013-08-05

**Authors:** Juhwan Kim, Miyoung Yang, Sung-Ho Kim, Jong-Choon Kim, Hongbing Wang, Taekyun Shin, Changjong Moon

**Affiliations:** 1 Departments of Veterinary Anatomy and Veterinary Toxicology, College of Veterinary Medicine and Animal Medical Institute, Chonnam National University, Gwangju, Republic of Korea; 2 Department of Physiology and Neurosceince Program, Michigan State University, East Lansing, Michigan, United States of America; 3 Department of Veterinary Anatomy, College of Veterinary Medicine, Jeju National University, Jeju, Republic of Korea; Federal University of Rio de Janeiro, Brazil

## Abstract

Trimethyltin (TMT) is an organotin compound with potent neurotoxic effects characterized by neuronal destruction in selective regions, including the hippocampus. Glycogen synthase kinase-3 (GSK-3) regulates many cellular processes, and is implicated in several neurodegenerative disorders. In this study, we evaluated the therapeutic effect of lithium, a selective GSK-3 inhibitor, on the hippocampus of adult C57BL/6 mice with TMT treatment (2.6 mg/kg, intraperitoneal [i.p.]) and on cultured hippocampal neurons (12 days *in vitro*) with TMT treatment (5 µM). Lithium (50 mg/kg, i.p., 0 and 24 h after TMT injection) significantly attenuated TMT-induced hippocampal cell degeneration, seizure, and memory deficits in mice. In cultured hippocampal neurons, lithium treatment (0–10 mM; 1 h before TMT application) significantly reduced TMT-induced cytotoxicity in a dose-dependent manner. Additionally, the dynamic changes in GSK-3/β-catenin signaling were observed in the mouse hippocampus and cultured hippocampal neurons after TMT treatment with or without lithium. Therefore, lithium inhibited the detrimental effects of TMT on the hippocampal neurons *in vivo* and *in vitro*, suggesting involvement of the GSK-3/β-catenin signaling pathway in TMT-induced hippocampal cell degeneration and dysfunction.

## Introduction

Trimethyltin (TMT), an organotin compound, has potent neurotoxic effects characterized by neuronal destruction in selective regions such as the limbic system [1]–[3]. Humans accidentally exposed to TMT develop a syndrome characterized by seizures, disorientation, confusion, memory deficits and aggressiveness [1], [4]. In experimental animals, exposure to TMT induces neurotoxicity due to the initial oxidative burden in select regions [5], [6]. TMT-induced selective neuronal cell death and neuroinflammation contribute to neurodegeneration [7]. Several *in vivo* and *in vitro* studies demonstrated that c-Jun N-terminal kinase signaling, cyclooxygense-2, caspases-3/-8 and diverse proinflammatory cytokines are activated by TMT treatment and might be involved in the pathological mechanism of TMT-induced brain injury [8]–[11]. In mice, TMT treatment causes seizures, hyperactivity, memory deficits, and neuronal cell loss, especially in the hippocampal dentate gyrus (DG) [12], [13]. Recently, several studies suggested the phosphoinositol 3-kinase (PI3K)/Akt pathway to be a target for neuroprotection in TMT-induced central nervous system (CNS) injury [6], [14], [15]. Thus, TMT-induced neurotoxicity is regarded as a useful model for the study of neurodegenerative diseases and hippocampal dysfunction, such as Alzheimer’s disease (AD) [7]. However, the precise mechanism underlying TMT-induced neuronal cell death remains unclear.

Glycogen synthase kinase-3 (GSK-3) is a multifunctional serine/threonine (Ser/Thr) kinase initially reported to be a regulator of glycogen metabolism [16]. GSK-3 is comprised of two isoforms, GSK-3α and GSK-3β, which both play a pivotal role in regulating many processes such as cellular structure, function and survival. GSK-3 is regulated primarily by inhibitory serine phosphorylation *via* the PI3K/Akt signaling pathway and/or Wnt signaling pathway [17]–[19]. β-catenin is a key downstream molecule of the GSK-3 signaling and plays an important role in neuroprotection [20]–[22]. Several studies implicated dysregulation of GSK-3 activity in CNS disorders such as AD, schizophrenia and bipolar disorders [23]–[25]. Recently, lithium, a selective GSK-3 inhibitor, has been shown to ameliorate neurodegeneration, neuroinflammation, and behavioral disability following traumatic brain injury (TBI) [26], [27] and kainate-induced neurotoxicity *in vivo* and *in vitro* [28], [29]. Furthermore, Fabrizi et al. [30] reported that several autophagy activators, including lithium, attenuate TMT-induced neurotoxicity in cultured hippocampal neurons. However, the involvement of the GSK-3/β-catenin signaling pathway in TMT-induced neurodegeneration is unclear.

In the present study, we elucidated the changes in the GSK-3/β-catenin signaling pathway in TMT-induced hippocampal degeneration and the neuroprotective effects of lithium on TMT-induced neurotoxicity *in vivo* and *in vitro* to elucidate the possible role of GSK-3 signaling in chemical-induced neurodegeneration.

## Results

Figure l shows a schematic diagram of the procedures used for *in vivo* tests evaluating the effect of lithium treatment on TMT-induced neurodegeneration and behavioral disability.

**Figure 1:**
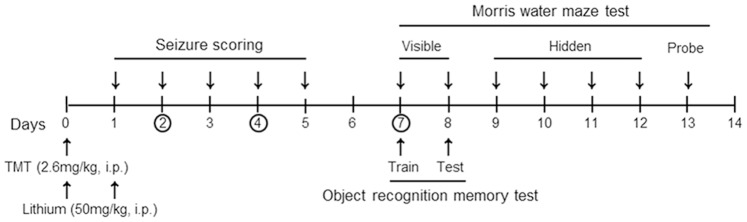
Schematic diagram of drug treatment, tissue preparation and behavioral tests. Mice were treated with lithium chloride (50 mg/kg, i.p.) 0 and 24 h after TMT (2.6 mg/kg, i.p.) injection. Then, mice were monitored and seizure scored for 5 consecutive days. Learning and memory tests (novel object recognition memory and Morris water maze) were performed after disappearance of TMT-induced seizures (7 days post-treatment). Circles indicate the time-points at which were sacrificed and tissue was sampled.

### TMT Induced the Change of GSK-3/β-catenin Signaling in the Hippocampus

**Figure 2:**
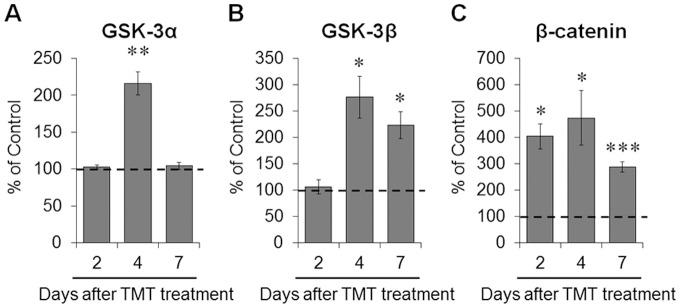
TMT administration induced alteration of GSK-3 activity in the mouse hippocampus. Mice were treated with TMT (2.6 mg/kg, i.p.) and hippocampi were dissected at various time points for Western blot analysis. (A) Bar graphs show a significant increase in GSK-3α (Ser^21^) phosphorylation in the hippocampus 4 days post-treatment. (B) Bar graphs show a significant increase in GSK-3β (Ser^9^) phosphorylation in the hippocampus 4 and 7 days post-treatment. To quantify the inhibitory phosphorylation of either GSK-3α or GSK-3β, phosphorylated forms were normalized to either total GSK-3α or GSK-3β. (C) Bar graphs show a significant increase in β–catenin expression in the hippocampus 2, 4 and 7 days post-treatment. For normalization of β–catenin expression, the membranes were reprobed with β-actin antibody. Immunoblot images for phospho-GSK-3α (Ser^21^), total GSK-3α, phospho-GSK-3β (Ser^9^), total GSK-3β, β–catenin and β-actin are shown in the Supporting Information (Fig. S1). The data are reported as the means±SEM (*n* = 3 per group). *p<0.05, **p<0.01, ***p<0.001 *vs.* controls. Cont, controls; TMT, TMT-treated mice.

To determine the effect of TMT treatment on the GSK-3 pathway, the inhibitory serine phosphorylation of GSK-3 and the β-catenin expression levels in hippocampal extracts prepared 2, 4 and 7 days post-treatment (*n* = 3 mice in each time-point) were assessed by Western blot analysis (Fig. 2 and Fig. S1). TMT treatment led to significant increases in the inhibitory phosphorylation of GSK-3α (Ser^21^) 4 days post-treatment (*p*<0.01 *vs.* controls; Fig. 2A), and GSK-3β (Ser^9^) 4 and 7 days post-treatment (*p*<0.05 *vs.* controls; Fig. 2B). The treatment also markedly increased the level of β-catenin expression 2 (*p*<0.05 *vs.* controls), 4 (*p*<0.05 *vs.* controls) and 7 days post-treatment (*p*<0.001 *vs.* controls) (Fig. 2C).

Consistent with the Western blotting results, the phosphorylated GSK-3α (Ser^21^) and GSK-3β (Ser^9^) and β-catenin expression levels, measured by immunohistochemistry, were localized primarily in *Cornu Ammonis* (CA) 1 pyramidal and dentate gyrus (DG) granule neurons in the hippocampus, and markedly increased in the granular cell layer (GCL) of the DGs 4 days after TMT treatment (Fig. S2).

### Lithium Treatment Rescued TMT-induced Seizure

**Figure 3:**
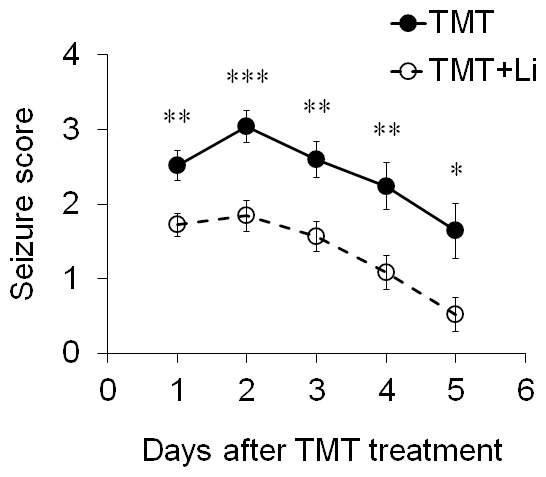
Lithium treatment significantly ameliorated TMT-induced clinical symptoms in mice. Lithium (50 mg/kg, i.p.) injection 0 and 24 h after TMT (2.6 mg/kg, i.p.) rescued TMT-induced seizure behaviors (*n* = 25 mice per group). The data are reported as the means±SEM. *p<0.05, **p<0.01, ***p<0.001 *vs.* TMT-treated mice. TMT, TMT-treated mice; TMT+Li, TMT+lithium-treated mice.

TMT exposure causes symptoms such as tremor, seizure and aggressive behavior in mice (Fig. 3). However, the TMT-induced seizure score in lithium-treated mice was significantly lower than that in TMT-treated controls (*n* = 25 mice per group; Fig. 3). The seizure behaviors in TMT-treated controls and TMT+lithium-treated mice had disappeared on day 6 after TMT treatment. Table 1 summarizes the effect of lithium on the clinical symptoms of TMT-treated mice.

**Table 1:** Effect of Lithium Chloride on the Clinical Symptoms of Mice after TMT Injection.

Treatment	Incidence of death	Average of max.seizure score
TMT	5/25	3.2±0.23
TMT+Lithium	1/25	1.9±0.21^a^

Mice were treated with lithium chloride (50 mg/kg, i.p.) 0 and 24 h after a single administration of TMT, and their clinical symptoms monitored by seizure scoring for 5 days after TMT administration. Data are expressed as means±SEM.

a, ***p<0.001 *vs.* vehicle-treated controls.

### Lithium Treatment Ameliorated TMT-induced Memory Deficits in Mice

We first assessed mouse basal locomotor activity 7 days after TMT treatment in a novel environment by open-field analysis (*n* = 10 mice per group). The open-field analysis quantified the overall activity that reflects the motivation and performance of the mice. The control, lithium-, TMT-, and TMT+lithium-treated mice showed comparable ambulatory movement counts, moving distances, ambulatory movement times, and resting times, with no significant differences observed in any group (Fig. S3).

Next, we assessed recognition memory in mice (*n* = 9 mice per group) by a sensitive hippocampus-dependent paradigm, object recognition memory [31], [32]. Control, lithium-, TMT-, and TMT+lithium-treated mice displayed equal preference for the two objects during training 7 days after TMT treatment (Fig. S4A). Additionally, the total number of interactions during training was 13.78±0.55 in vehicle-treated controls, 14.10±1.40 in lithium-treated mice, 13.33±0.78 in TMT-treated mice and 14.11±0.63 in TMT+lithium-treated mice (Fig. S4B). There were no significant differences among groups, suggesting that mice had comparable attention, motivation and visual perception.

**Figure 4:**
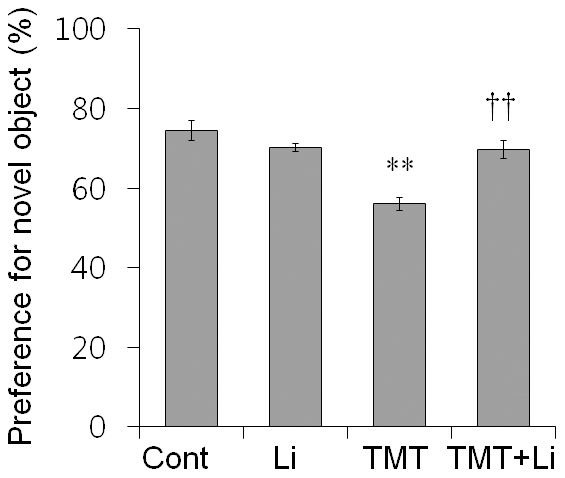
Lithium treatment significantly ameliorated TMT-induced deficits in novel object recognition memory in mice. Mice were treated with lithium (50 mg/kg, i.p.) 0 and 24 h after TMT (2.6 mg/kg, i.p.) administration, and evaluated using the novel object recognition memory test (*n* = 9 mice per group). During the test, TMT-treated mice showed a significantly decreased preference for the novel object; however, TMT+lithium-treated mice showed a preference for the novel object comparable to control mice. The data are reported as the means±SEM. **p<0.01 *vs.* controls. ^††^ *p*<0.01 *vs.* TMT-treated mice. Cont, controls; Li, lithium-treated mice; TMT, TMT-treated mice; TMT+Li, TMT+lithium-treated mice.

During the test 24 h after training, one conditioned old object was replaced with a novel object. If mice retained memory for the old objects, they would show preference for the novel object. The preferences (mean±SEM) toward a novel object were 74.44±2.49% in controls, 70.24±0.97% in lithium-treated mice, 55.98±1.61% in TMT-treated mice, and 70.59±3.60% in TMT+lithium-treated mice (Fig. 4A). Thus, TMT-treated mice showed significant deficits in novel object recognition (*p*<0.01 *vs.* controls), which were ameliorated by lithium treatment (*p*<0.01 *vs.* TMT-treated mice).

**Figure 5:**
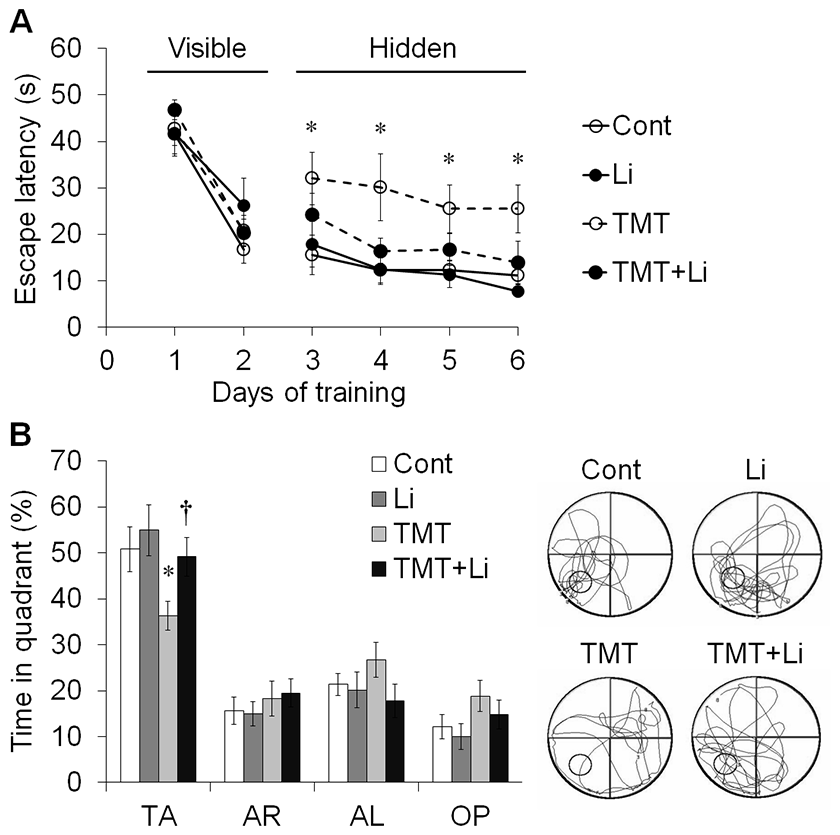
Lithium treatment significantly improved spatial memory deficits induced by TMT administration in mice. Mice were treated with lithium (50 mg/kg, i.p.) 0 and 24 h after TMT (2.6 mg/kg, i.p.) administration, and tested using the Morris water maze from 7 to 13 days post-treatment (*n* = 10 mice/group). (A) In the visible platform training from day 1 to 2 (7–8 days post-treatment), mice in different groups showed similar latency before escaping to find the visible platform. In the hidden platform training from day 3 to 6 (9–12 days post-treatment), TMT-treated mice showed a significant increase in escape latency. (B) Bar graphs in the left panel show that TMT+lithium-treated mice spent a significantly longer time in the target quadrant compared to TMT-treated mice during the probe trial on day 7 (13 days post-treatment). Right panel shows representative swim paths in the water maze during the probe trial. The data are reported as the means±SEM. *p<0.05 *vs.* controls. ^†^ *p*<0.05 *vs.* TMT-treated mice. TA, Target quadrant; AL, adjacent left quadrant; OP, opposite quadrant; Cont, controls; Li, lithium-treated mice; TMT, TMT-treated mice; TMT+Li, TMT+lithium-treated mice.

We further examined hippocampus-dependent spatial memory in mice (*n* = 10 mice per group) using the Morris water maze test [32]. In the visible platform training, mice learned to find the escape platform which had an attached visual cue. There was no significant difference between groups in escape latency during visible platform training (Fig. 5A). However, TMT-treated mice showed significantly longer escape latency compared to controls during the hidden platform training (*p*<0.05; Fig. 5A).

During the probe test 24 h after training, the percentages of time spent in the target quadrant were 50.82±4.92% in controls, 54.93±5.57% in lithium-treated mice, 36.23±3.14% in TMT-treated mice and 49.13±4.17% in TMT+lithium-treated mice. Thus, TMT-treated mice showed significant deficits in spatial memory (*p*<0.05 *vs.* controls), which was rescued by lithium treatment (*p*<0.05 *vs.* TMT-treated mice) (Fig. 5B).

### Lithium Treatment Ameliorated TMT-induced Neuronal Cell Death in the Hippocampus

According to behavioral data, lithium treatment ameliorates TMT-induced hippocampal dysfunction, suggesting that lithium decreases TMT-induced neuronal cell death in the hippocampal DG. Therefore, we first performed hematoxylin and eosin staining 2, 4, and 7 days post-treatment. There were no significant differences in hippocampal structure among the groups under low magnification (Fig. S5). However, remarkable granular cell death, characterized by eosinophilic cytoplasm, nuclear pyknosis, nuclear karyolysis and cell loss, was evident in the hippocampal DG under high magnification at each time-point after TMT treatment. Lithium treatment reduced the TMT-induced granular cell death in the hippocampal DG (Fig. S5).

**Figure 6:**
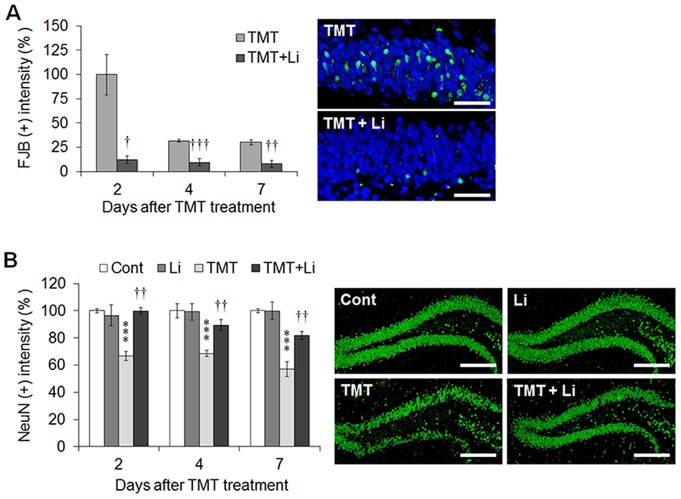
Lithium significantly reduced TMT-induced neuronal cell death in the mouse hippocampus. (A) Bar graphs in the left panel show that lithium administration significantly reduced the density of Fluoro-jade B (FJB)-positive degenerating neurons in the dentate gyrus (DG) of adult mouse hippocampus 2, 4 and 7 days after TMT treatment. Representative photomicrographs (right panels) of FJB (Green) and DAPI stained granular cells (Blue) in the DG of adult hippocampus in TMT-treated mice (upper panel) and TMT+lithium-treated mice (lower panel). Scale bars = 30 µm. (B) Bar graphs in the left panels show that lithium treatment significantly reduced neuronal cell loss in the DG of mice 2, 4 and 7 days after TMT treatment. Representative photomicrographs (right panels) of NeuN immunoreactivity in the DG of adult hippocampus of control (Cont), lithium-treated (Li), TMT-treated (TMT) and TMT+lithium-treated mice (TMT+Li). Scale bars = 200 µm. The data are reported as the means±SEM (*n* = 3 per group). *p<0.05, **p<0.01, ***p<0.001 *vs.* controls. ^†^ *p*<0.05, ^††^ *p*<0.01, ^†††^ *p*<0.001 *vs.* TMT-treated mice. Cont, controls; Li, lithium-treated mice; TMT, TMT-treated mice; TMT+Li, TMT+lithium-treated mice.

We additionally performed Fluoro-jade B (FJB) staining and NeuN immunostaining to detect neuronal degeneration and survival, respectively, to clarify the protective effects of lithium on TMT-induced neuronal cell death in the hippocampal DG (*n* = 3 mice in each time point). Semi-quantitative analysis of the FJB-positive intensity showed that lithium treatment resulted in a significant decrease in degenerative neurons in TMT-treated mice 2 (*p*<0.05 *vs.* TMT-treated mice), 4 (*p*<0.001 *vs.* TMT-treated mice) and 7 days (*p*<0.01 *vs.* TMT-treated mice) after TMT administration (Fig. 6A). Semi-quantitative analysis of NeuN-positive intensity showed that TMT treatment resulted in significant neuronal cell loss in mice 2, 4 and 7 days after TMT administration (*p*<0.001 *vs.* controls). However, lithium treatment significantly reduced TMT-induced neuronal cell loss 2, 4 and 7 days (*p*<0.01 *vs.* TMT-treated mice) after TMT administration (Fig. 6B).

### Lithium Treatment Inhibited the TMT-induced Change of GSK-3/β-catenin Signaling in the Mouse Hippocampus

**Figure 7:**
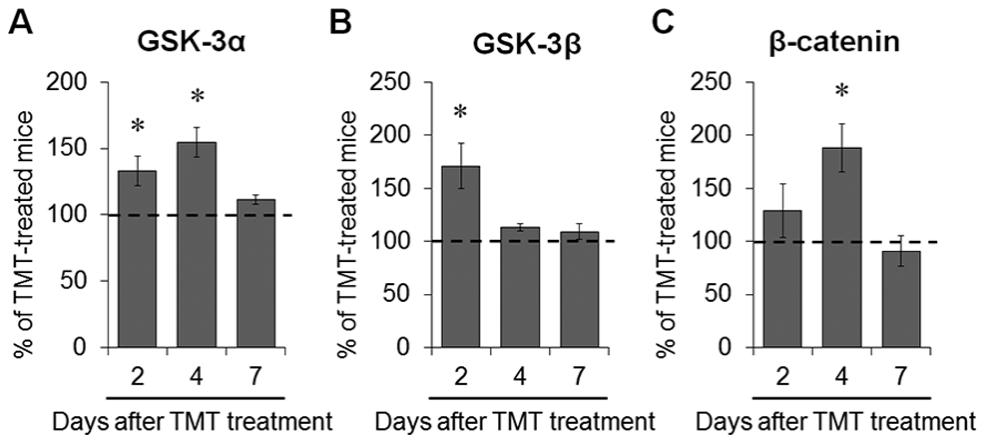
Lithium treatment inhibited the GSK-3 signaling pathway in the mouse hippocampus after TMT treatment. Mice were treated with lithium chloride (50 mg/kg, i.p.) 0 and 24 h after TMT (2.6 mg/kg, i.p.) administration and hippocampi were dissected at various time–points for Western blot analysis. (A) Bar graphs show significant increases in the inhibitory phosphorylation of GSK-3α (Ser^21^) in the hippocampus 2 and 4 days post-treatment. (B) Bar graphs show a significant increase in the inhibitory phosphorylation of GSK-3β (Ser^9^) in the hippocampus 2 days post-treatment. To quantify the inhibitory phosphorylation of either GSK-3α or GSK-3β, phosphorylated forms were normalized to either total GSK-3α or GSK-3β. (C) Bar graphs show a significant increase in β–catenin expression in the hippocampus 4 days post-treatment. For normalization of β–catenin expression, the membranes were reprobed with β-actin antibody. Immunoblot images for phospho-GSK-3α (Ser^21^), total GSK-3α, phospho-GSK-3β (Ser^9^), total GSK-3β, β–catenin and β-actin are shown in the Supporting Information (Fig. S1). The data are reported as the means±SEM (*n* = 3 per group). *p<0.05 *vs.* TMT-treated mice. TMT, TMT-treated mice; TMT+Li, TMT+lithium-treated mice.

To confirm that lithium treatment inhibits GSK-3 activity in the hippocampus of TMT-treated mice, we evaluated the inhibitory phosphorylation of GSK-3α (Ser^21^) and GSK-3β (Ser^9^) and the level of β-catenin expression in the hippocampus by Western blotting (*n* = 3 mice in each time-point; Fig. S1). Lithium treatment significantly increased the inhibitory phosphorylation of GSK-3α (Ser^21^) 2 and 4 days after TMT treatment (*p*<0.05 *vs.* TMT-treated mice; Fig. 7A), and GSK-3β (Ser^9^) 2 days after TMT treatment (*p*<0.05 *vs.* TMT-treated mice; Fig. 7B) in the hippocampus. Lithium treatment also increased the level of β-catenin expression 4 days after TMT treatment (*p*<0.05 *vs.* TMT-treated mice; Fig. 7C).

### Lithium Treatment Protected Hippocampal Cultured Neurons against TMT-induced Neurotoxicity

**Figure 8:**
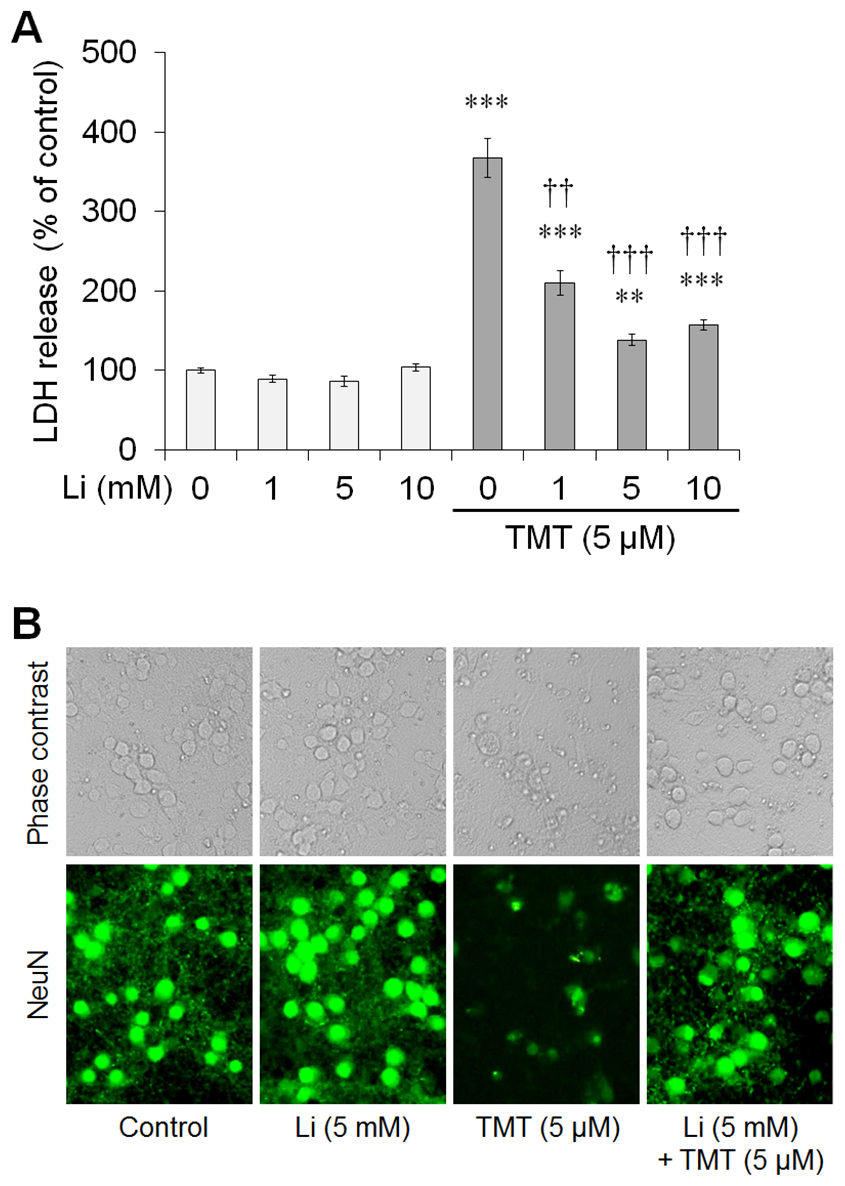
Lithium treatment significantly inhibited TMT-induced cytotoxicity. (A) Pre-treatment of lithium 1 h before TMT treatment (5 µM) significantly inhibited LDH release in hippocampal neurons with all treatment doses (0–10 mM) compared with TMT-treated cultures. (B) Representative photomicrographs of phase contrast (upper panels) and NeuN immunofluorescence (lower panels) in hippocampal neurons. The data are reported as the means±SEM. *n* = 6 cultures per condition. **p<0.01, ***p<0.001 *vs.* controls; ^††^ *p*<0.01, ^†††^ *p*<0.001 *vs.* TMT-treated cultures (5 µM). Cont, controls; TMT, TMT-treated cultures TMT+Li, TMT+lithium-treated cultures.

Based on our previous study [33], we tested whether lithium treatment rescued TMT-induced cytotoxicity in mature hippocampal cells at 12 days *in vitro* (DIV) using lactate dehydrogenase (LDH) release assays. TMT (5 µM) increased LDH release from cultured hippocampal neurons 24 h post-treatment (*n* = 6 cultures per condition; *p*<0.001 *vs.* controls; Fig. 8A). However, TMT-induced cytotoxicity was significantly inhibited by lithium (1–10 mM) in a dose-dependent manner (*n* = 6 cultures per condition; Fig. 8A).

To confirm the protective effect of lithium on TMT-induced neurotoxicity, we performed NeuN immunostaining in primary hippocampal cultures 24 h post-treatment. Lithium treatment remarkably reduced TMT-induced neuronal cell death (*n* = 3 cultures per condition; Fig. 8B). Thus, consistent with the *in vivo* data, lithium significantly rescued neuronal cell death induced by TMT treatment in mature hippocampal cells *in vitro*.

### Lithium Treatment Inhibited the Effect of TMT on GSK-3/β-catenin Signaling in Hippocampal Cultured Neurons

**Figure 9:**
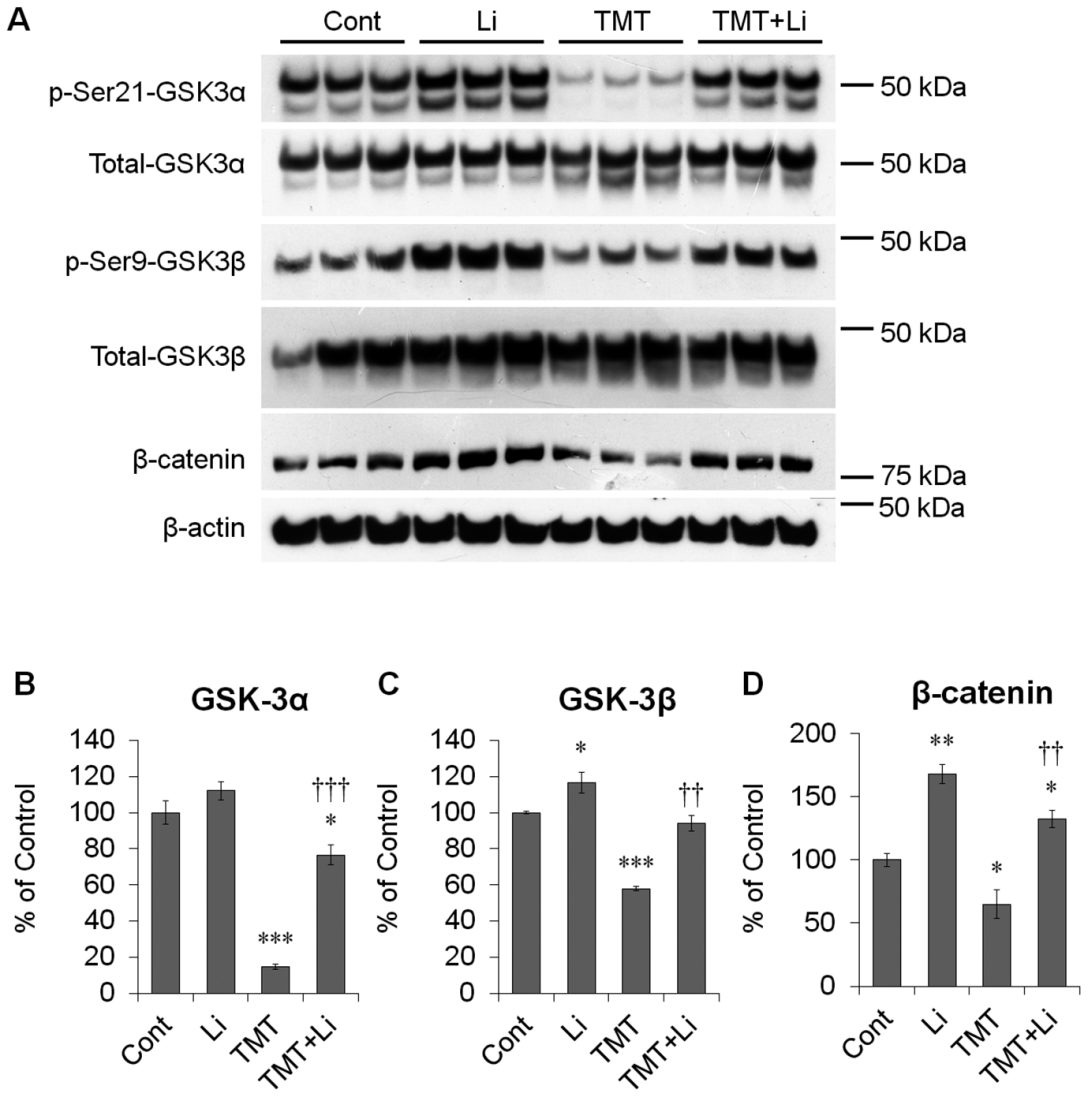
Lithium treatment inhibited the GSK-3 pathway in hippocampal neurons after TMT treatment. At 12 DIV, hippocampal neurons were pre-treated with lithium (5 mM) 1 h before TMT treatment (5 µM). (A) Representative immunoblots for phospho-GSK-3α (Ser^21^), total GSK-3α (∼51 kDa), phospho-GSK-3β (Ser^9^), total GSK-3β (∼46 kDa), β-catenin (∼92 kDa) and β-actin (∼45 kDa). (B–D) Bar graphs show significant decreases in the inhibitory phosphorylation of GSK-3α (Ser^21^) and GSK-3β (Ser^9^), and β-catenin expression of in TMT-treated cultures. However, lithium pre-treatment considerably increased the expression levels compared to TMT-treated cultures. To quantify the inhibitory phosphorylation of either GSK-3α or GSK-3β, phosphorylated forms were normalized to either total GSK-3α or GSK-3β. For normalization of β-catenin expression, the membranes were reprobed with anti-β-actin antibody. The data are reported as the means±SEM. *n* = 6 cultures per condition. *p<0.05, **p<0.01, ***p<0.001 *vs.* controls. ^††^ *p*<0.01, ^†††^ *p*<0.001 *vs.* TMT-treated cultures. Cont, controls; Li, lithium-treated cultures; TMT, TMT-treated cultures; TMT+Li, TMT+lithium-treated cultures.

To determine if GSK-3 activity is altered by TMT exposure and inhibited by lithium preconditioning in hippocampal cultured neurons, we assessed the inhibitory phosphorylation of GSK-3α (Ser^21^) and GSK-3β (Ser^9^) and the level of β-catenin expression in mature hippocampal cells at 12 DIV by Western blotting. TMT treatment significantly decreased the inhibitory phosphorylation of GSK-3α (Ser^21^) and GSK-3β (Ser^9^) and the level of β-catenin expression 24 h post-treatment. However, lithium treatment markedly increased the inhibitory phosphorylation of GSK-3α (Ser^21^) and GSK-3β (Ser^9^) and the level of β-catenin expression (Fig. 9).

## Discussion

Our data demonstrate that inhibition of GSK-3 signaling attenuates TMT-induced neurodegeneration in adult mice, as reflected by neuronal cell death in the hippocampal DG, clinical symptoms characterized by tremor/seizure and memory deficit, and TMT-induced cytotoxicity in primary hippocampal cultured neurons. Furthermore, in present study, TMT and/or lithium modulated the GSK-3/β-catenin signaling in hippocampal neurons *in vivo* and *in vitro*.

GSK-3, a multifunctional Ser/Thr kinase that regulates many cellular processes, is regulated by the Wnt and/or PI3K/Akt signaling pathways through distinct mechanisms [34], [35]. In the Wnt signaling pathway, a multi-protein destruction complex consisting of the Wnt receptor, Frizzled low-density lipoprotein receptor-related protein 5/6, axin, adenomatous polyposis coli, β-catenin and casein kinase-1, regulates GSK-3 activity by means of protein–protein interactions [36], [37]. Moreover, GSK-3 activity is regulated by direct phosphorylation of Akt, cyclic-AMP-dependent protein kinase, p70 ribosomal S6 kinase, and p90 ribosomal S6 kinase [38]–[41]. GSK-3-mediated Ser33/37 phosphorylation of β-catenin is reduced by inactivation of either Wnt or PI3K/Akt signaling, leading to its accumulation in the cytosol and transcriptional activation [42]–[44]. Furthermore, since β-catenin is a key mediator of the GSK-3 signaling pathway and plays an important role in neuroprotection, it is regarded as a potent survival factor [45], [46]. Therefore, β-catenin accumulation may be used as an indicator of inhibition of GSK-3 activity. In the present study, TMT exposure dramatically altered the inhibitory phosphorylation of GSK-3 and accumulation of β-catenin in the hippocampus, which is a selective TMT target region in mice, suggesting the GSK-3/β-catenin signal pathway to be associated with TMT-induced neurodegeneration.

Several studies have suggested dysregulation of GSK-3 activity to be involved in neurodegeneration by neurotoxic agents *in vivo* and *in vitro* [34], [47], [48]. Recently, a microarray study revealed that GSK-3α gene expression increased in the hippocampus of rats 3 days after TMT treatment [49]. We hypothesized that TMT treatment might induce upregulation of GSK-3 activity in the hippocampus, because the present study showed TMT-induced neuronal cell death in the mouse hippocampus. However, the *in vivo* data showed that TMT treatment significantly increased the inhibitory serine phosphorylations of GSK-3α (Ser^21^) 2 days post-treatment and GSK-3β (Ser^9^) 4 and 7 days post-treatment in the adult mouse hippocampus. We also observed accumulation of β-catenin in the mouse hippocampus 2, 4 and 7 days post-treatment. These results are consistent with the kainate-induced neurodegeneration model [29], in which the kainate induced an increase of inhibitory phosphorylation of GSK-3 in hippocampal neurons as a compensatory survival response against kainate toxicity. Consequently, we suggest that the increases in the inhibitory serine phosphorylation of GSK-3 and the accumulation of β-catenin in the mouse hippocampus elicited by TMT treatment might mediate neuronal cell survival against TMT-induced neurotoxicity.

Lithium was recently shown to directly inhibit GSK-3 activity by competition with Mg^2+^ for binding to its catalytic site and indirectly by inhibitory serine phosphorylation of GSK-3 *via* Akt pathways [50], [51]. Previous studies revealed that lithium provides neuroprotective effects by inhibition of GSK-3 activity in various animal models of neurodegenerative disorders including TBI, AD, and ischemic stroke [26], [52], [53]. Lithium has also been shown to have an anticonvulsant effect on chemical-induced seizure behavior [54], [55]. Furthermore, lithium improves the cognitive impairment induced by streptozotocin, mild TBI, and brain ischemia [26], [56]–[59]. In the present study, lithium treatment significantly ameliorated TMT-induced seizure behavior and cognitive impairments in the object recognition memory and Morris water maze paradigms.

Accumulating *in vivo* and *in vitro* evidence suggests that inhibition of GSK-3 activity by lithium may contribute to its therapeutic effects [26], [60], [61]. Previous studies revealed that lithium treatment reduces neuronal cell death, inflammation and oxidative stress, and inhibits apoptotic pathways dependent on GSK-3 inhibition [57], [62]–[64]. Pre- or post-treatment with lithium reduces apoptotic cell death in an AD model and protects primary-cultured neurons from glutamate-induced excitotoxicity *via* inhibition of GSK-3 [65], [66]. In the present study, lithium treatment significantly reduced the histopathological lesions–as shown by hematoxylin and eosin staining–the density of FJB-positive degenerating neurons, and the loss of NeuN-positive neurons in the hippocampal DG in TMT-treated mice. Therefore, inhibition of GSK-3 in the mouse hippocampus by lithium treatment may reduce neuronal cell death following TMT treatment. Furthermore, lithium treatment significantly increased the inhibitory serine phosphorylation of GSK-3 and the level of β-catenin expression in the mouse hippocampus post-treatment. These findings suggest that inhibition of GSK-3 is crucial for the therapeutic effects of lithium on TMT-induced neurotoxicity.

In addition, our *in vitro* data showed that inhibition of GSK-3 activity by lithium treatment markedly protected hippocampal neurons against TMT-induced cytotoxicity. Pre-treatment of lithium attenuates chemical-induced cytotoxicity through the GSK-3 signaling pathway [30], [67], and increases the inhibitory phosphorylation of GSK-3 induced by neurotoxic agents in cultured cells [67], [68]. In the present *in vitro* study, Western blot analysis showed that TMT treatment significantly decreased the level of the inhibitory phosphorylation of GSK-3α (Ser^21^) and GSK-3β (Ser^9^) and the level of β-catenin expression 24 h post-treatment. Conversely, lithium treatment markedly increased the inhibitory phosphorylation of GSK-3α (Ser^21^) and GSK-3β (Ser^9^) and the level of β-catenin expression 24 h post-treatment, suggesting that lithium-induced accumulation of β-catenin may be related to its crucial neuroprotective role against TMT-induced neurodegeneration. Therefore, consistent with our initial hypothesis, the *in vitro* data suggest that GSK-3 activity is closely related to TMT-induced neurotoxicity in mouse hippocampal neurons.

In conclusion, TMT exposure dramatically altered the GSK-3/β-catenin signaling pathway in the mouse hippocampus, and GSK-3 inhibition significantly reduced TMT-induced behavioral and histological changes in the hippocampus, as well as cytotoxicity in hippocampal cultured neurons. These *in vivo* and *in vitro* findings suggest that the GSK-3 signal pathway is associated with TMT-induced hippocampal neurodegeneration. Furthermore, the use of a selective GSK-3 inhibitor, lithium, may have therapeutic effects on chemical-induced neurodegeneration, including TMT-induced neurotoxicity.

## Materials and Methods

### Animals

Male C57BL/6 mice, 8- to 9-weeks-old, were obtained from a specific-pathogen-free colony at Orient Bio, Inc. (Seoul, Korea). The Institutional Animal Care and Use Committee of Chonnam National University approved the protocols used in this study (CNU IACUC-YB-2012-18) and the animals were cared for in accordance with the Chonnam National University Guide for the Care and Use of Laboratory Animals.

### Drug Treatment and Tissue Sampling

TMT (Wako, Osaka, Japan) and lithium chloride (Sigma-Aldrich, St. Louis, MO, USA) were dissolved in sterilized 0.9% saline. To assess the time-dependent effects of TMT on the GSK-3 signal pathway in mouse hippocampus, mice were sacrificed and the brains were dissected at 2, 4 and 7 days after a single intraperitoneal (i.p.) injection of vehicle (0.9% saline) or 2.6 mg/kg TMT (*n* = 6 mice per group). All the animals were killed by decapitation. The hippocampal samples and brains were stored at –70°C until used for Western blot analysis and stored in 30% sucrose after fixation in 4% paraformaldehyde in phosphate-buffered saline (PBS, pH 7.4) for immunohistochemistry, respectively.

To assess the effect of lithium on TMT-induced seizures, lithium chloride (50 mg/kg, i.p.) or vehicle (0.9% saline, i.p.) was administered to mice at 0 and 24 h after an administration of 2.6 mg/kg TMT (*n* = 25 mice per group), and seizure behaviors were observed for 5 consecutive days after TMT treatment. To assess the effects of lithium on TMT-induced memory deficits, hippocampus-dependent memory tests (the object recognition memory test [*n* = 9 mice/group] and the Morris water maze test [*n* = 10 mice per group]) were performed 7 days post-treatment, at which time seizure behavior had disappeared.

To assess the effects of lithium treatment on inhibitory phosphorylation of GSK-3 in the hippocampus, mice were sacrificed 2, 4, and 7 days post-treatment (*n* = 6 mice per group). The samples were embedded in paraffin wax after fixation in 4% paraformaldehyde in PBS (pH 7.4) using routine protocols (*n* = 3 mice per group) and stored at –70°C for biochemical analysis (*n* = 3 mice per group).

### Antibodies

Polyclonal rabbit anti-phospho-GSK-3α (Ser^21^), polyclonal rabbit anti-phospho-GSK-3β (Ser^9^), monoclonal rabbit anti-active β-catenin, monoclonal rabbit anti-β-catenin, monoclonal rabbit anti-GSK-3α, and monoclonal rabbit anti-GSK-3β antibodies were purchased from Cell Signaling Technology (Beverly, MA, USA). Monoclonal mouse anti-neuronal nuclei (NeuN) antibody (Millipore, Temecula, CA, USA) was used to detect neurons. Monoclonal mouse anti-beta actin was purchased from Sigma-Aldrich. For immunoblot analysis, horseradish peroxidase (HRP)-conjugated anti-rabbit IgG and anti-mouse IgG were obtained from Vector Laboratories (Burlingame, CA, USA). Immunofluorescent staining was performed using a fluorescein isothiocyanate (FITC)-conjugated secondary antibody (Vector).

### Western Blot Analysis

Mouse hippocampi were individually immersed immediately in buffer H (50 mM β-glycerophosphate, 1.5 mM ethylene glycol tetraacetic acid, 0.1 mM Na_3_VO_4_, 1 mM dithiothreitol, 10 µg/mL aprotinin, 2 µg/mL pepstatin, 10 µg/mL leupeptin,1 mM phenylmethanesulfonylfluoride, pH 7.4), and sonicated for 8 s. SDS sample buffer (×4) was added to each homogenized sample, and the samples were heated at 100°C for 10 min. The samples were then separated by 10% SDS-PAGE (Bio-Rad, Hercules, CA, USA). Medium was completely removed from hippocampal cell culture by aspiration and SDS sample buffer (×4) was added to each culture. Cells from each culture were scraped and sonicated for 4 s. The samples were heated at 100°C for 10 min, and then separated by 10% SDS-PAGE.

The resolved proteins were transferred to a nitrocellulose membrane blocked with 1% normal goat serum (Vector) and 0.5% fetal bovine serum (Sigma-Aldrich) in PBS containing 0.1% Tween 20 (PBS-T, pH 7.4) for 1 h at room temperature (RT). The membranes were then incubated with primary antibodies, including rabbit anti-phospho-GSK-3α (1∶1,000 dilution), anti-phospho-GSK-3β (1∶1,000 dilution), or anti-β-catenin (1∶1,000 dilution), in PBS-T (0.2% Tween 20 in PBS) overnight at 4°C. After extensive washing and incubation with HRP-conjugated anti-rabbit antibody (1∶10,000 dilution; Thermo Fisher Scientific, Inc., Rockford, IL, USA), signals were visualized using a chemiluminescence kit (SuperSignal® West Pico; Thermo Fisher Scientific, Inc.). To quantify the inhibitory phosphorylation of either GSK-3α or GSK-3β, the membranes were reprobed with an antibody to either total GSK-3α (1∶1,000 dilution) or GSK-3β (1∶1,000 dilution). For the normalization of β-catenin expression, the membranes were reprobed with antibody to β-actin (1∶20,000 dilution). Several exposure times were used to obtain the signals in a linear range. The bands were quantified using the Scion Image Beta 4.0.2 for Windows XP software (Scion, Frederick, MD, USA).

### Immunohistochemistry

Detailed in the Supporting Information (Text S1).

### Open-field Test

Detailed in the Supporting Information (Text S1).

### Seizure Scoring

Tremor/seizure tests were performed in brightly lit arenas (40×40 cm, 250 lux). Behavioral changes were scored as follows: (1) aggression; (2) weak tremor; (3) systemic tremor; (4) tremor and spasmodic gait; and (5) death [33], [69].

### Object Recognition Memory Test

The object-recognition memory test was used to examine hippocampus-dependent memory [31], [32]. The test was similar to a test described previously [70]. Briefly, two randomly selected, different-shaped objects were presented to each mouse for 10 min during training. Next, 24 h after training, another pair of objects (one old object and one novel object) was presented to the trained mice. If, for example, cube- and pyramid-shaped objects were presented during training, then a cylinder-shaped object was used as a novel object during testing. The interactions of the mouse with each object, including approaches and sniffing, were scored. If the mouse remembered an old object, preference toward the novel object was demonstrated during testing. The preference percentage was defined as the number of interactions for a specific object divided by the total number of interactions for both objects.

### Morris Water Maze Test

The Morris water maze test was used to assess hippocampus-dependent spatial memory [71]. Mice were individually trained in a circular pool (100 cm diameter, 30 cm height) filled with water maintained at 25°C and made opaque using a non-toxic washable white paint. The maze was located in a lit room with extramaze cues. The escape platform (10 cm diameter) was placed in the center of a designated quadrant of the pool with its top positioned 1 cm below the water surface. Mice were not allowed to swim in the pool before training. During the visible platform training, the platform was marked by a flag (5 cm tall). Mice were subjected to six trials daily for 2 days. The six trials were divided into two blocks with 1-h intervals. Three trials per block were conducted at 10-min intervals. Each trial lasted for 60 s unless mice reached the platform. The time elapsed until the mouse reached and land on the platform was scored as the escape latency. If the mouse failed to find the platform within 60 s, it was gently navigated to the platform by hand. Whether mice found or failed to find the platform within 60 s, they were allowed to stay on the platform for 30 s. After the visible platform training, the mice were further subjected to the hidden platform training, during which the platform was placed 1 cm below the opaque water. The position of the platform was fixed and starting positions were used pseudo-randomly between trials. Mice were subjected to four trials at 1-h intervals daily for 4 consecutive days. Probe trials were performed 24 h after the hidden platform training. The platform was removed from its previous location and mice were allowed to swim in the pool for 1 min. The time spent in quadrant, number of crossings for the location of the hidden platform and swim speed were measured by video-based tracking system (SMART VIDEO-TRACKING; Panlab, Barcelona, Spain).

### FJB Staining

FJB (a high-affinity fluorescent marker for the localization of neuronal degeneration) histofluorescent staining was performed according to a method described previously [72]. In brief, the sections were first transferred to a solution of 0.06% potassium permanganate and then to 0.0004% FJB (Millipore) staining solution. After washing, the sections were counterstained with DAPI before being mounted. The FJB-stained sections were examined by immunofluorescence microscopy using a BX-40 apparatus with an eXcope X3 digital camera.

### Immunofluorescence

NeuN immunoreactivity was examined using immunofluorescence labeling of the same section 2, 4 and 7 days post-treatment and on primary hippocampal culture 24 h post-treatment. In brief, the sections and hippocampal cultures were blocked with 10% normal goat serum (Vector) in PBS-T, and then incubated overnight at 4°C with mouse anti-NeuN antibody (1∶50 dilution) in PBS-T for neuron detection. After incubation with primary antibody, sections were exposed to FITC-labeled anti-mouse IgG (1∶50 dilution) for 1 h at RT. After washing, immunofluorescence-stained sections and hippocampal cultures were examined by immunofluorescence microscopy using a BX-40 apparatus with an eXcope X3 digital camera and a Leica DM IRB apparatus (Leica Microsystems, Wetzlar, Germany) with a ProgRes® CFscan digital camera (Jenoptik, Jena, Germany), respectively.

### Semi-quantitative Analyses of FJB Intensity and NeuN-positive Reactions

The FJB intensity and NeuN immunoreactivity in the hippocampus were quantified using the ImageJ software (NIH, Bethesda, MD, USA). Mouse brains were sampled at approximately 2.12 mm behind the bregma. A standardized analysis area that contained 5-µm-thick coronal sections in a 1-in-10 series of sections representing the rostral/mid-hippocampus was used. For each mouse, three non-overlapping sections were analyzed, one from each of the three regions of the hippocampus (∼50 µm apart). All positively labeled cells in the DG were quantified. The mean value of positive intensity in the three sections of each mouse was taken as *n* = 1. Intensities were expressed as means±SEM (*n* = 3).

### Primary Hippocampal Cell Culture and Drug Treatment

The primary hippocampal cell culture method has been described previously [73]. Briefly, hippocampi were dissected from C57BL/6 mice pups at 17–18 gestational days, and prepared for culturing. After dissection, tissues were chopped and digested with 10 units/mL papain (Worthington, Freehold, NJ, USA) and 100 units/mL DNase I (Roche, Basel, Switzerland) in dissociation buffer at 37°C for 30 min. The digestion was triturated with Neurobasal A medium (Invitrogen, Carlsbad, CA, USA). The cells were seeded at a density of 0.3×10^6^ cells/well on poly-D-lysine hydrobromide (150 µg/mL; Sigma-Aldrich)-coated 24-well plates (NUNC™; Thermo Fisher Scientific, Inc.). Neurobasal A was replaced 1 h after plating with growth medium including Neurobasal A, 1× B27 supplement (Invitrogen), 100 units/mL penicillin, 0.1 mg/mL streptomycin, and 0.5 mM glutamine (Invitrogen). All cultures were maintained at 37°C and 5% CO_2_. Primary hippocampal cultured neurons at 12 DIV were treated with 0, 1, 5, or 10 µM of TMT and assayed 24 h post-treatment. To evaluate the cytoprotective effects of lithium on mature hippocampal neurons with TMT, lithium (0–10 mM) was added 1 h before TMT treatment (*n* = 6 cultures per condition).

### Cytotoxicity Evaluation

Cytotoxicity in hippocampal cultured neurons was evaluated using a LDH release assay. A commercially available LDH-cytotoxicity assay kit from Biovision (Mountain View, CA, USA) was used as recommended by the manufacturer. The optical densities at 490 nm were determined using a microplate reader (Emax, Molecular Devices).

### Statistical Analysis

The data are reported as means±SEM and were analyzed by one-way analysis of variance (ANOVA) followed by the Student–Newman–Keuls *post hoc* test for multiple comparisons. In all analyses, *p*<0.05 was taken to indicate statistical significance.

## Supporting Information

Figure S1 Immunoblot images for phospho-GSK-3α (Ser^21^), total GSK-3α (∼51 kDa), phospho-GSK-3β (Ser^9^), total GSK-3β (∼46 kDa), β–catenin (∼92 kDa) and β-actin (∼45 kDa) in the mouse hippocampus 2 (A), 4 (B) and 7 days (C) after TMT (2.6 mg/kg, i.p.) treatment. Cont, controls; Li, lithium-treated mice; TMT, TMT-treated mice; TMT+Li, TMT+lithium-treated mice.
(TIF)

Figure S2 **Immunohistochemical assays of phospho-GSK-3α (Ser^21^), phospho-GSK-3β (Ser^9^), and active β-catenin expression levels in the adult mouse hippocampus after TMT treatment.** The expression levels of phospho-GSK-3α (Ser^21^), phospho-GSK-3β (Ser^9^), and active β-catenin in the mouse hippocampus, especially the dentate granule cell blades, increased significantly at day 4 post-treatment. Cont, controls; TMT, TMT-treated mice. Scale bars represent 50 µm.
(TIF)

Figure S3 **TMT treatment did not alter the basal locomotor activity in open field analysis.** Mice were treated with lithium chloride (50 mg/kg, i.p.) 0 and 24 h after TMT administration (2.6 mg/kg, i.p.), and the basal locomotor activity was examined using the open-field test 7 days after TMT treatment (*n* = 10 per group). (A) Each group showed comparable movement distance (*p = *0.793 [Li], *p = *0.944 [TMT], *p = *0.992 [TMT+Li] *vs.* controls). (B) Each group showed similar ambulatory movement time (*p = *0.109 [Li], *p = *0.839 [TMT], *p = *0.544 [TMT+Li] *vs.* controls). (C) Each group showed similar movement episodes (*p = *0.974 [Li], *p = *0.413 [TMT], *p = *0.702 [TMT+Li] *vs.* controls). (D) Each group showed similar resting time (*p = *0.513 [Li], *p = *0.701 [TMT], *p = *0.369 [TMT+Li] *vs.* controls). The data are reported as the means±SEM.
(TIF)

Figure S4 **The preference for the two objects and the total number of interactions during training 7 days after TMT treatment.** (A) The control, lithium-treated, TMT-treated, and TMT+lithium-treated mice showed equal preference for the two objects during training. (B) There was no significant difference in the interaction with the two training objects during training. The data are reported as the means±SEM. Cont, controls; Li, lithium-treated mice; TMT, TMT-treated mice; TMT+Li, TMT+lithium-treated mice.
(TIF)

Figure S5 **Histopathological findings in the hippocampus of mice after TMT and TMT+lithium treatment.** Low magnification images of adult mouse hippocampus (upper panels at each day post-treatment) and high magnification images of the DG in the hippocampus (lower panels at each day post-treatment) at 2, 4 and 7 days post-treatment. CA, *cornu amonis*; GCL, granular cell layer; DG, dentate gyrus. The sections were stained with hematoxylin and eosin. Scale bars represent 300 µm (upper panels at each day post-treatment) and 30 µm (lower panels at each day post-treatment).
(TIF)

Text S1 **Supporting information for the Materials and Methods.**
(DOC)
