# Supplementary figures and images for: Possible Role of the Glycogen Synthase Kinase-3 Signaling Pathway in Trimethyltin-Induced Hippocampal Neurodegeneration in Mice
Source: PLoS One. 2013 Aug 5;8(8):e70356. doi: 10.1371/journal.pone.0070356 (PMC3734066; doi:10.1371/journal.pone.0070356)

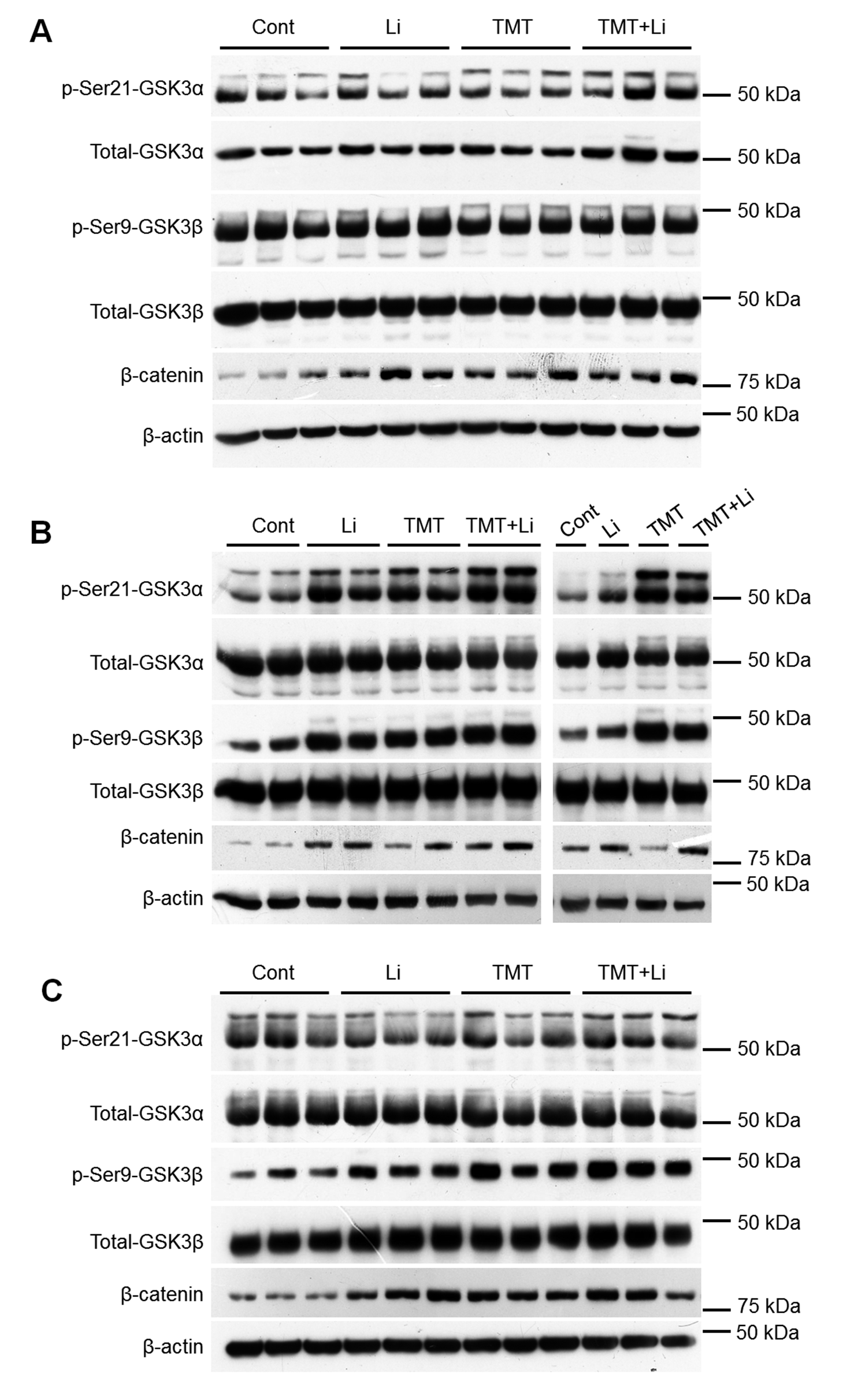

Supplement: Figure S1 — Immunoblot images for phospho-GSK-3α (Ser21), total GSK-3α (∼51 kDa), phospho-GSK-3β (Ser9), total GSK-3β (∼46 kDa), β–catenin (∼92 kDa) and β-actin (∼45 kDa) in the mouse hippocampus 2 (A), 4 (B) and 7 days (C) after TMT (2.6 mg/kg, i.p.) treatment. Cont, controls; Li, lithium-treated mice; TMT, TMT-treated mice; TMT+Li, TMT+lithium-treated mice. (TIF) [file pone.0070356.s001.tif]

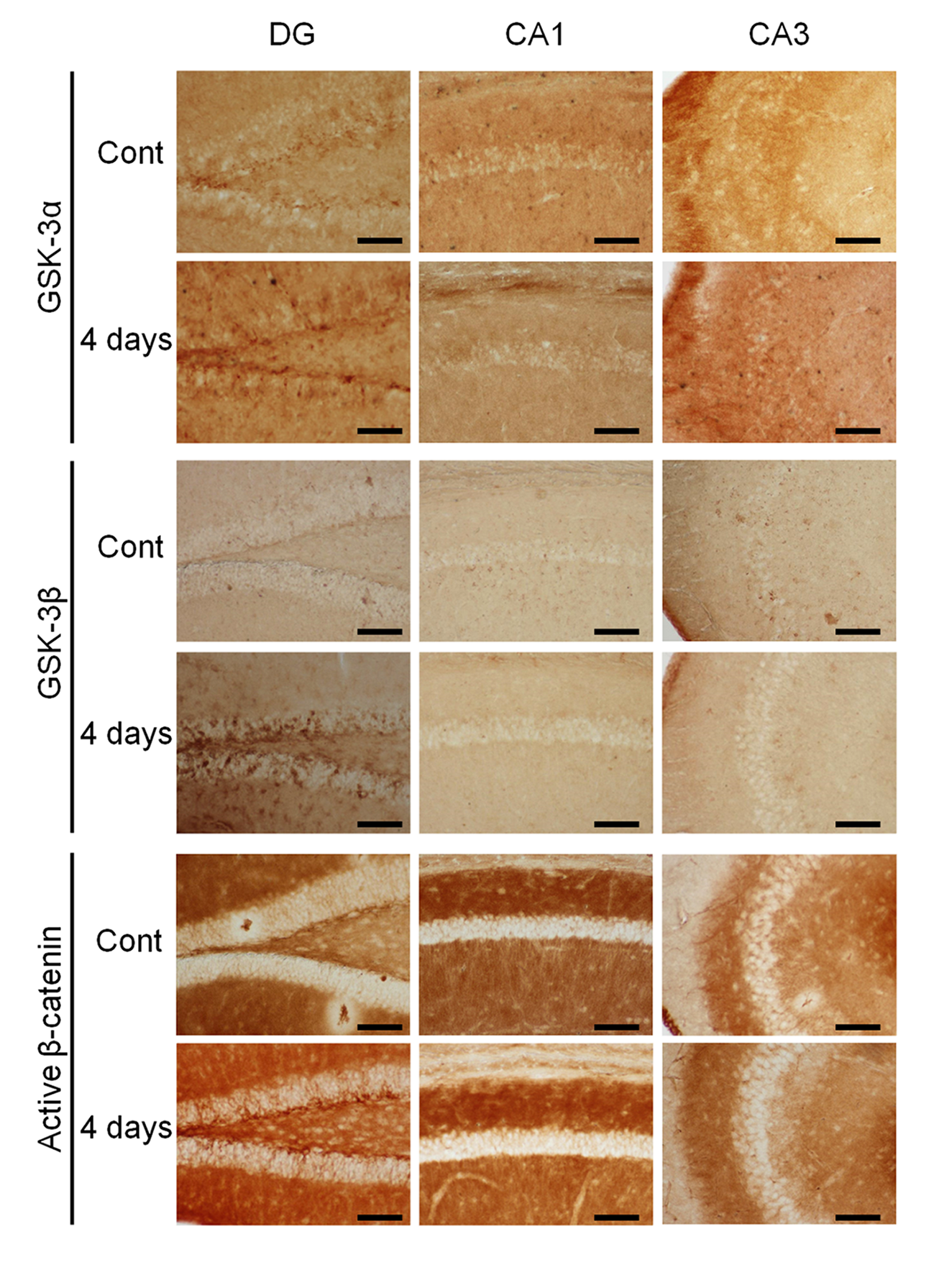

Supplement: Figure S2 — Immunohistochemical assays of phospho-GSK-3α (Ser21), phospho-GSK-3β (Ser9), and active β-catenin expression levels in the adult mouse hippocampus after TMT treatment. The expression levels of phospho-GSK-3α (Ser21), phospho-GSK-3β (Ser9), and active β-catenin in the mouse hippocampus, especially the dentate granule cell blades, increased significantly at day 4 post-treatment. Cont, controls; TMT, TMT-treated mice. Scale bars represent 50 µm. (TIF) [file pone.0070356.s002.tif]

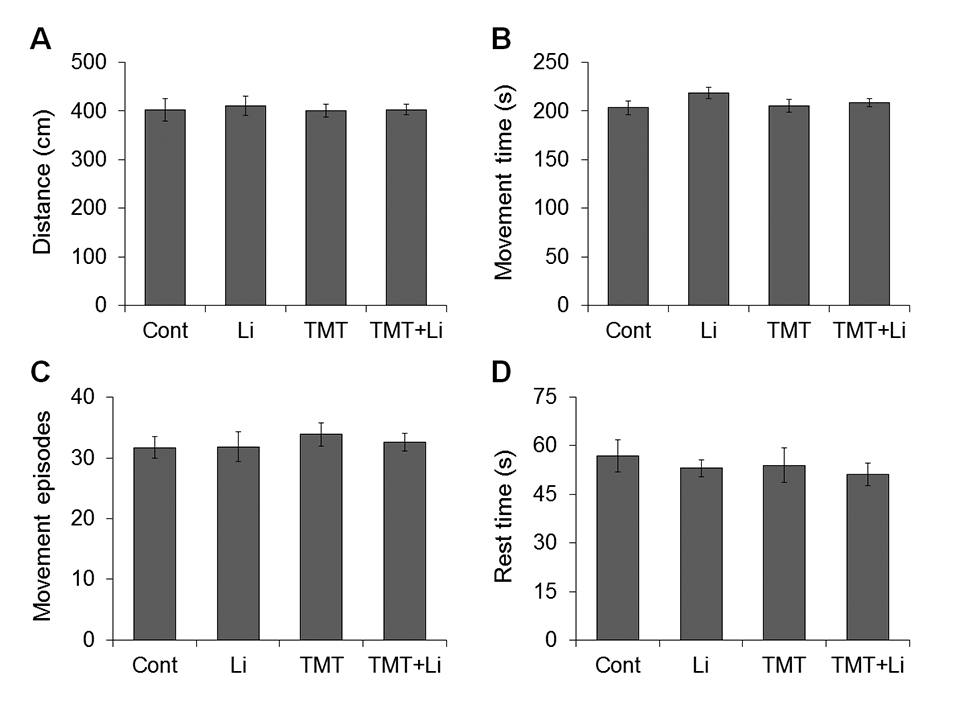

Supplement: Figure S3 — TMT treatment did not alter the basal locomotor activity in open field analysis. Mice were treated with lithium chloride (50 mg/kg, i.p.) 0 and 24 h after TMT administration (2.6 mg/kg, i.p.), and the basal locomotor activity was examined using the open-field test 7 days after TMT treatment (n = 10 per group). (A) Each group showed comparable movement distance (p = 0.793 [Li], p = 0.944 [TMT], p = 0.992 [TMT+Li] vs. controls). (B) Each group showed similar ambulatory movement time (p = 0.109 [Li], p = 0.839 [TMT], p = 0.544 [TMT+Li] vs. controls). (C) Each group showed similar movement episodes (p = 0.974 [Li], p = 0.413 [TMT], p = 0.702 [TMT+Li] vs. controls). (D) Each group showed similar resting time (p = 0.513 [Li], p = 0.701 [TMT], p = 0.369 [TMT+Li] vs. controls). The data are reported as the means±SEM. (TIF) [file pone.0070356.s003.tif]

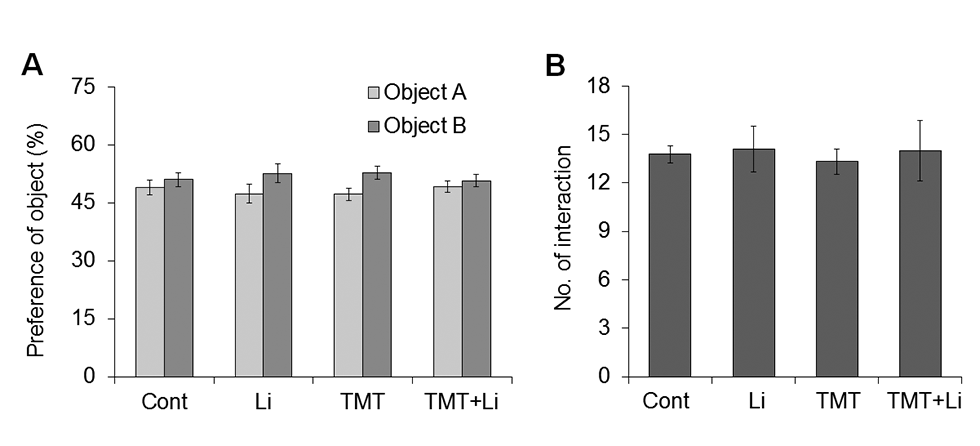

Supplement: Figure S4 — The preference for the two objects and the total number of interactions during training 7 days after TMT treatment. (A) The control, lithium-treated, TMT-treated, and TMT+lithium-treated mice showed equal preference for the two objects during training. (B) There was no significant difference in the interaction with the two training objects during training. The data are reported as the means±SEM. Cont, controls; Li, lithium-treated mice; TMT, TMT-treated mice; TMT+Li, TMT+lithium-treated mice. (TIF) [file pone.0070356.s004.tif]

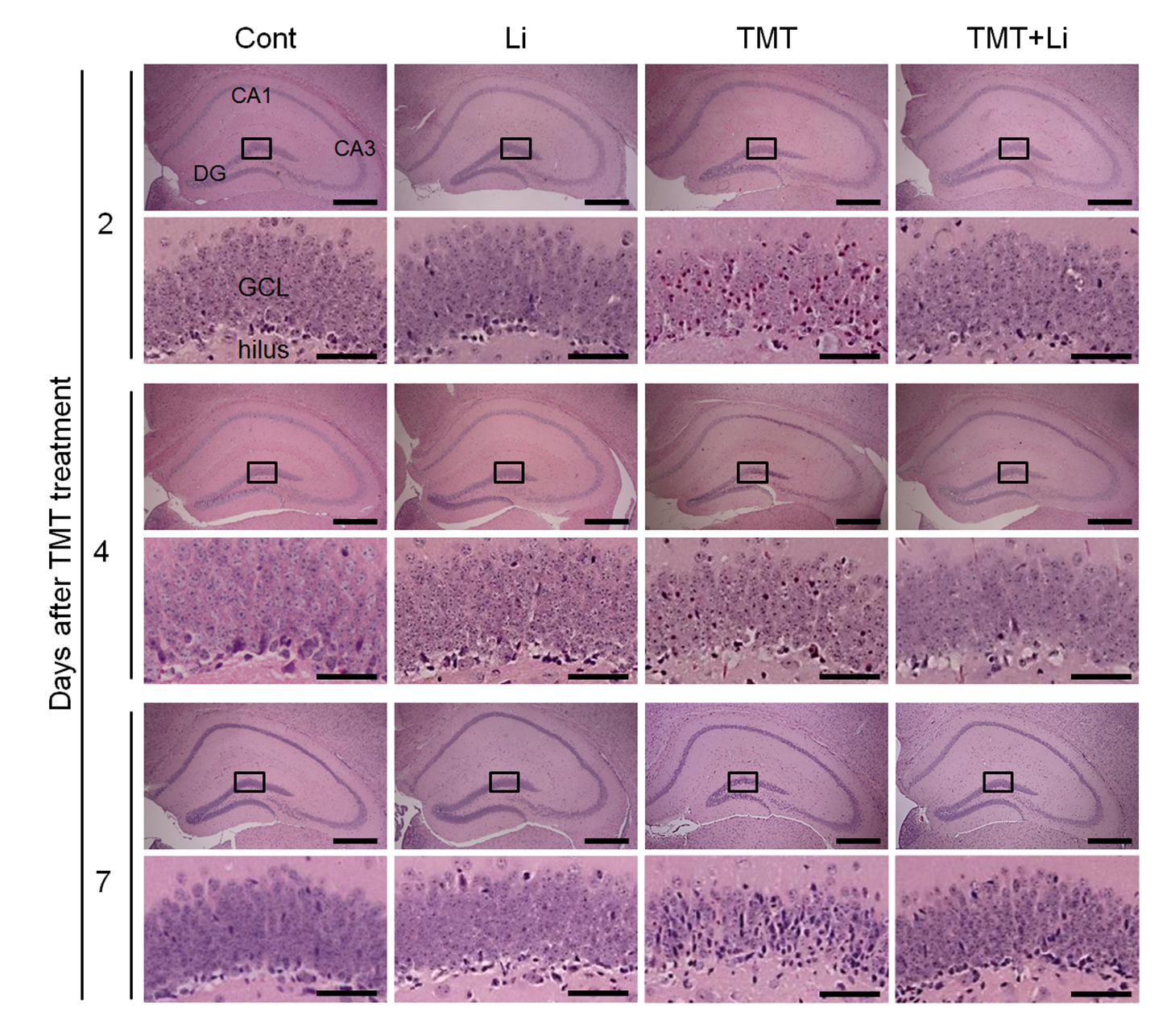

Supplement: Figure S5 — Histopathological findings in the hippocampus of mice after TMT and TMT+lithium treatment. Low magnification images of adult mouse hippocampus (upper panels at each day post-treatment) and high magnification images of the DG in the hippocampus (lower panels at each day post-treatment) at 2, 4 and 7 days post-treatment. CA, cornu amonis; GCL, granular cell layer; DG, dentate gyrus. The sections were stained with hematoxylin and eosin. Scale bars represent 300 µm (upper panels at each day post-treatment) and 30 µm (lower panels at each day post-treatment). (TIF) [file pone.0070356.s005.tif]
