# Supplementary material for: Possible Role of the Glycogen Synthase Kinase-3 Signaling Pathway in Trimethyltin-Induced Hippocampal Neurodegeneration in Mice
Source: PLoS One. 2013 Aug 5;8(8):e70356. doi: 10.1371/journal.pone.0070356 (PMC3734066; doi:10.1371/journal.pone.0070356)
Supplement: Text S1 — Supporting information for the Materials and Methods. (DOC) [file pone.0070356.s006.doc]

**Supporting Information for the Materials and Methods**

Immunohistochemistry

Free-floating sagittal brain sections were cut at a 30-μm thickness using a sliding microtome (SM2010R; Leica Microsystem, Wetzlar, Germany). Mouse brains were sectioned laterally at approximately 1.8 mm, starting from the medial border of the hippocampus and extending laterally to the start of the ventral hippocampus. For immunohistochemistry, the sagittal sections were deactivated with endogenous peroxidase (5% hydrogen peroxide in methyl alcohol and 0.2% Triton X-100) and then blocked with 10% normal goat serum (Vector) in PBS-T. Sections were then incubated with primary antibodies, including rabbit anti-phospho-GSK-3α (1:500 dilution), anti-phospho-GSK-3β (1:500 dilution), or anti-active β-catenin (1:500 dilution), in PBS-T for 6 days at 4°C. After three washes, the sections were reacted with biotinylated goat anti-rabbit IgG (Vector ABC Elite Kit) for 1 h at RT. After three washes, the sections were incubated for 1 h at RT with an avidin-biotin peroxidase complex (Vector ABC Elite Kit) prepared according to the manufacturer’s instructions. After three washes, the peroxidase reaction was developed for 3 min using a diaminobenzidine substrate (DAB kit; Vector Laboratories) prepared according to the manufacturer’s instructions. As a control, the primary antibodies were omitted from a few test sections in each experiment. The immunohistochemistry-stained specimens were observed using a BX-40 apparatus (Olympus, Tokyo, Japan) with an eXcope X3 digital camera (DIXI Optics, Daejeon, South Korea).

Open-field test

Open-field analysis was used to measure the activities of mice in a novel environment post-treatment. Parameters including ambulatory movement count, total moving distance, ambulatory movement time, and resting time were determined by the TruScan Photo Beam Activity System (Coulbourn Instruments, Whitehall, PA, USA)

Object recognition memory test

The object-recognition memory test was used to examine hippocampus-dependent memory. The test was similar to a test described previously. Brieﬂy, two randomly selected, different-shaped objects were presented to each mouse for 10 min during training. Next, 24 h after training, another pair of objects (one old object and one novel object) was presented to the trained mice. If, for example, cube- and pyramid-shaped objects were presented during training, then a cylinder-shaped object was used as a novel object during testing. The interactions of the mouse with each object, including approaches and snifﬁng, were scored. If the mouse remembered an old object, preference toward the novel object was demonstrated during testing. The preference percentage was deﬁned as the number of interactions for a speciﬁc object divided by the total number of interactions for both objects.
